# Clinical features of squamous cell lung cancer with anaplastic lymphoma kinase (ALK)-rearrangement: a retrospective analysis and review

**DOI:** 10.18632/oncotarget.25257

**Published:** 2018-05-08

**Authors:** Junko Watanabe, Shinsaku Togo, Issei Sumiyoshi, Yukiko Namba, Kentaro Suina, Takafumi Mizuno, Kotaro Kadoya, Hiroaki Motomura, Moe Iwai, Tetsutaro Nagaoka, Shinichi Sasaki, Takuo Hayashi, Toshimasa Uekusa, Kanae Abe, Yasuo Urata, Fuminori Sakurai, Hiroyuki Mizuguchi, Shunsuke Kato, Kazuhisa Takahashi

**Affiliations:** ^1^ Department of Respiratory Medicine, Juntendo University Faculty of Medicine & Graduate School of Medicine, Tokyo 113-8431, Japan; ^2^ Research Institute for Diseases of Old Ages, Juntendo University Graduate School of Medicine, Tokyo 113-8431, Japan; ^3^ Department of Respiratory Medicine, Juntendo University Urayasu Hospital, Chiba 279-0001, Japan; ^4^ Junior Resident of Juntendo University Hospital, Tokyo 113-8431, Japan; ^5^ Department of Pharmacy, Juntendo University School of Medicine, Tokyo 113-8431, Japan; ^6^ Department of Pathology, Juntendo University School of Medicine, Tokyo 113-8431, Japan; ^7^ Department of Pathology, Labor Health and Welfare Organization Kanto Rosai Hospital, Kanagawa 211-8510, Japan; ^8^ Oncolys BioPharma, Inc, Minato-ku, Tokyo 105-0001 Japan; ^9^ Laboratory of Biochemistry and Molecular Biology, Graduate School of Pharmaceutical Sciences, Osaka University, Osaka 565-0871, Japan; ^10^ Department of Medical Oncology, Juntendo University School of Medicine, Tokyo 113-8431, Japan

**Keywords:** anaplastic lymphoma kinase rearrangement, circulating tumor cells, epithelial-mesenchymal transition, squamous cell lung cancer, TelomeScan

## Abstract

Anti-anaplastic lymphoma kinase (ALK)-targeted therapy dramatically improves therapeutic responses in patients with ALK-rearranged lung adenocarcinoma (Ad-LC). A few cases of squamous cell lung carcinoma (Sq-LC) with ALK rearrangement have been reported; however, the clinicopathological features and clinical outcomes following treatment with ALK inhibitors are unknown. We addressed this in the present study by retrospectively comparing the clinical characteristics of five patients with ALK-rearranged Sq-LC with those of patients with ALK-rearranged Ad-LC and by evaluating representative cases of ALK inhibitor responders and non-responders. The prevalence of ALK rearrangement in Sq-LCs was 1.36%. Progression-free survival (PFS) after initial treatment with crizotinib was significantly shorter in Sq-LC than in Ad-LC with ALK rearrangement (*p* = 0.033). Two ALK rearrangements assayed by fluorescence *in situ* hybridization (FISH)-positive/immunohistochemistry-negative cases did not respond to crizotinb, and PFS decreased following alectinib treatment of ALK-rearranged Sq-LC (*p* = 0.045). A rebiopsy revealed that responders to ceritinib harbored the L1196M mutation, which causes resistance to other ALK inhibitors. However, non-responders were resistant to all ALK inhibitors, despite the presence of ALK rearrangement in FISH-positive circulating tumor cells and circulating free DNA and absence of the ALK inhibitor resistance mutation. These results indicate that ALK inhibitors remain a reasonable therapeutic option for ALK-rearranged Sq-LC patients who have worse outcomes than ALK-rearranged Ad-LC patients and that resistance mechanisms are heterogeneous. Additionally, oncologists should be aware of the possibility of ALK-rearranged Sq-LC based on clinicopathological features, and plan second-line therapeutic strategies based on rebiopsy results in order to improve patient outcome.

## INTRODUCTION

Oncogenic fusion of anaplastic lymphoma kinase (ALK) with echinoderm microtubule-associated protein-like 4 resulting from genetic rearrangement is observed in 5% of patients with lung adenocarcinoma (Ad-LC), who tend to be younger and never or light smokers and exhibit aggressive invasion [1]. Targeted anti-ALK therapy has been shown to elicit good responses and dramatically prolong overall survival (OS) in patients with non-small cell lung cancer (NSCLC) harboring ALK rearrangement. In fact, the U.S. Food and Drug Administration (FDA) has already approved three ALK inhibitors (crizotinib, ceritinib, and alectinib) for clinical trials in this patient population [2–7]. The estimated prevalence of ALK rearrangement in squamous cell lung carcinoma (Sq-LC) is thought to be as low as ∼0.2%–2.5% [9, 10]. Hence, ALK rearrangement screening by fluorescence *in situ* hybridization (FISH) and/or immunohistochemistry (IHC) is not routinely performed in patients with Sq-LC, leading to inadequate identification of the molecular tumor subtype, which can affect decisions regarding the best treatment options. ALK-rearranged Sq-LC is extremely rare, and has been reported only i nisolated cases; in these studies, the rearrangements were identified and responses to first-generation ALK inhibitors were reported. It is unknown whether ALK inhibitors are effective in patients with ALK-rearranged Sq-LC; moreover, the on- and off-target resistance mechanisms of ALK inhibitors remain unclear. On-target resistance to crizotinib has been observed in approximately one-third of patients with ALK-rearranged Ad-LC [11, 12]. Such mechanisms of resistance to ALK inhibitors have been classified as either on-target genetic alterations (e.g., secondary mutation conferring resistance to ALK inhibitors or *ALK* gene amplification) or off-target effects (e.g., upregulation of bypass signaling pathways such as epidermal growth factor receptor [EGFR] and its ligands transforming growth factor [TGF]-α and insulin-like growth factor receptor 1 [IGF1R]) [11, 13–15].

Here we report the first retrospective investigation of the clinical features and outcomes of ALK-rearranged Sq-LC and Ad-LC patients treated with ALK inhibitors at our hospital. We also reviewed previous case reports of ALK-rearranged Sq-LCs as well as two representative cases of responders and non-responders to three ALK inhibitors, including information from rebiopsies that were performed when the patients acquired resistance to previously administered ALK inhibitors.

## RESULTS

### Clinicopathological features of ALK-rearranged Sq-LC

Among the 221 patients with pathologically diagnosed Sq-LC, three (1.36%) had ALK rearrangement, consistent with previous reports [9, 10]. Among the 28 patients with ALK-rearranged NSCLC, three (10.7%) had ALK-rearranged Sq-LC; all other cases were Ad-LC. There were no significant differences in age and sex between patients with ALK-rearranged Sq-LC and Ad-LC. Four of five patients with ALK-rearranged Sq-LC were ex- or current smokers, and the prevalence of ALK rearrangement in NSCLC with smoking was higher among Sq-LC as compared to Ad-LC patients (80.0% vs. 68.0%). However, six of 15 (40%) previously reported cases of ALK-rearranged Sq-LC had a history of smoking (Tables 1 and 3). All patients with ALK-rearranged Sq-LC had previously undergone standard chemotherapy for Sq-LC prior to treatment with crizotinib and switched regimens when ALK rearrangement was detected, when they were diagnosed with progressive disease (PD) during ongoing chemotherapy, or when they showed severe adverse effects after detection of ALK rearrangement. All cases of stage IV ALK-rearranged Sq-LC showed increased expression of the Sq-LC-specific marker cytokeratin fragment (CYFRA; > 3.5 ng/ml); the CYFRA values of stage IV ALK-rearranged Sq-LC and Ad-LC were 20.2 ± 32.6 and 9.6 ± 3.6 U/ml, respectively. Interestingly, the levels of sialyl SSEA-1 antigen (SLX) (> 37 U/ml)—an Ad-LC-specific marker—were also increased in all cases of stage IV ALK-rearranged Sq-LC (496.3 ± 869.3 vs. 178.8 ± 313.6 U/ml for ALK-rearranged Ad-LC).

**Table 1:** Clinicopathological features of previously reported cases of squamous and adenosquamous cell lung carcinoma with anaplastic lymphoma kinase rearrangement

Author, year	Age/sex	Smoking history	Cancer type		IHC for Sq diagnosis				ALK rearrangement detection			ALK inhibitor	
		PY	Sq or AdSq	Stage	p40	p63	TTF-1	Napsin A	IHC	FISH	% Positive FISH	Regimen	PFS
Mamesaya N, et al. 2017 [19]	52/F	Never	Sq	IV	+	N/A	−	N/A	+	+	46	Alectinib	11>
Yamamoto Y, et al. 2016 [21]	76/M	20	Sq	IIIB	+	N/A	−	−	−	+	20	N/A	N/A
Wang W, et al. 2016 [22]	37/F	Never	Sq	IIIB	N/A	+	−	−	+	N/A	N/A	Crizotinib	9
Vergne F, et al. 2016 [23]	58/F	Never	Sq	IV	+	+	−		+	+	80	Crizotinib	7.1
Mikes RE, et al. 2015 [40]	36/M	Never	Sq	N/A	+	+	−	−	+	+	26	Crizotinib	3
Zhang Q, et al. 2015 [24]	55/F	Never	Sq	IV	+	+	−	−	+	N/A	N/A	Crizotinib	6
Tamiya A, et al. 2015 [31]	78/M	49	Sq	N/A	+	N/A	−	N/A	+	+	20	Alectinib	1.5
Takanashi Y, et al. 2015 [41]	60/M	36	Sq	IB	+	+	−	N/A	+	N/A	N/A	None	N/A
Wang Q, et al. 2014 [25]	55/F	Never	Sq	IV	N/A	+	−	−	+	+	15	Crizotinib	5.8
Dragnev KH, et al. 2013 [42]	61/M	Never	AdSq^a^	IIA	+	+	−	N/A	+	+	78.5	None	N/A
Kim H, et al. 2013 [36]	36/F	Never	Sq	IV	N/A	+	−	N/A	+	+	82	None	N/A
Alrifai D, et al. 2013 [43]	69/M	40	Sq	IIIA	N/A	+	−	N/A	N/A	+	N/A	None	N/A
Ochi N, et al. 2013 [44]	45/F	25	Sq	N/A	+	+	−	N/A	+	+	60%	N/A	N/A
Chaft JE, et al. 2012 [26]	58/M	< 1	AdSq^b^	IV	N/A	+	−	N/A	N/A	+	N/A	Crizotinib	13
Klempner SJ, et al. 2011 [45]	47/M	Never	AdSq	IIIA	N/A	+	+	N/A	N/A	+	N/A	N/A	N/A

AdSq, adenosquamous; ALK, anaplastic lymphoma kinase; F, female; FISH, fluorescence *in situ* hybridization; IHC, immunohistochemistry; M, male; N/A, not available; PFS, progression-free survival; PY, pack year; Sq, squamous.

^a^Only lymph node metastases showed lung adenocarcinoma (p40^−^/p63^−^/TTF-1^+^).

^b^Only lymph node metastases showed squamous cell lung carcinoma with ALK rearrangement (p63^+^/TTF-1^−^).

**Table 3:** Characteristics of patients with squamous cell lung cancer with ALK rearrangement

	Squamous cell carcinoma (including two AdSq cases)	Adenocarcinoma
Number of patients	5^a^	25
Year (mean ± SD [range])	54.8 ± 12.8 [36–65]	59.1 ± 13.1 [38–83]
Male/female	3/2	16/9
% male	60.0%	64.0%
Smoking rate (%)	4/5 (80.0)	17/25 (68.0)
I/II/III/IV	0/0/1/4	3/1/5/16^b^
Treatment history Number of patients		
First ALK inhibitor C/A/Ce	5/0/0	8/6/0
Second ALK inhibitor C/A/Ce	3/0/0	0/6/0
Third ALK inhibitor C/A/Ce	0/0/2	0/0/0
		
Previous chemo and chemo + rad		
Number of patients	5	7
ALK mutation detection		
Number of patients (%)		
IHC	3 (60.0)	21 (84.0)
FISH	5 (100.0)	24 (96.0)^c^

A, alectinib; C, crizotinib; Ce: ceritinib; chemo, chemotherapy; FISH, fluorescence *in situ* hybridization; IHC, immunohistochemistry; rad: radiation therapy.

^a^Two patients were added from Urayasu Hospital for statistical analysis.

^b^Two patients underwent an operation; four relapsed after operation.

^c^One case was indeterminate.

**Figure 1:**
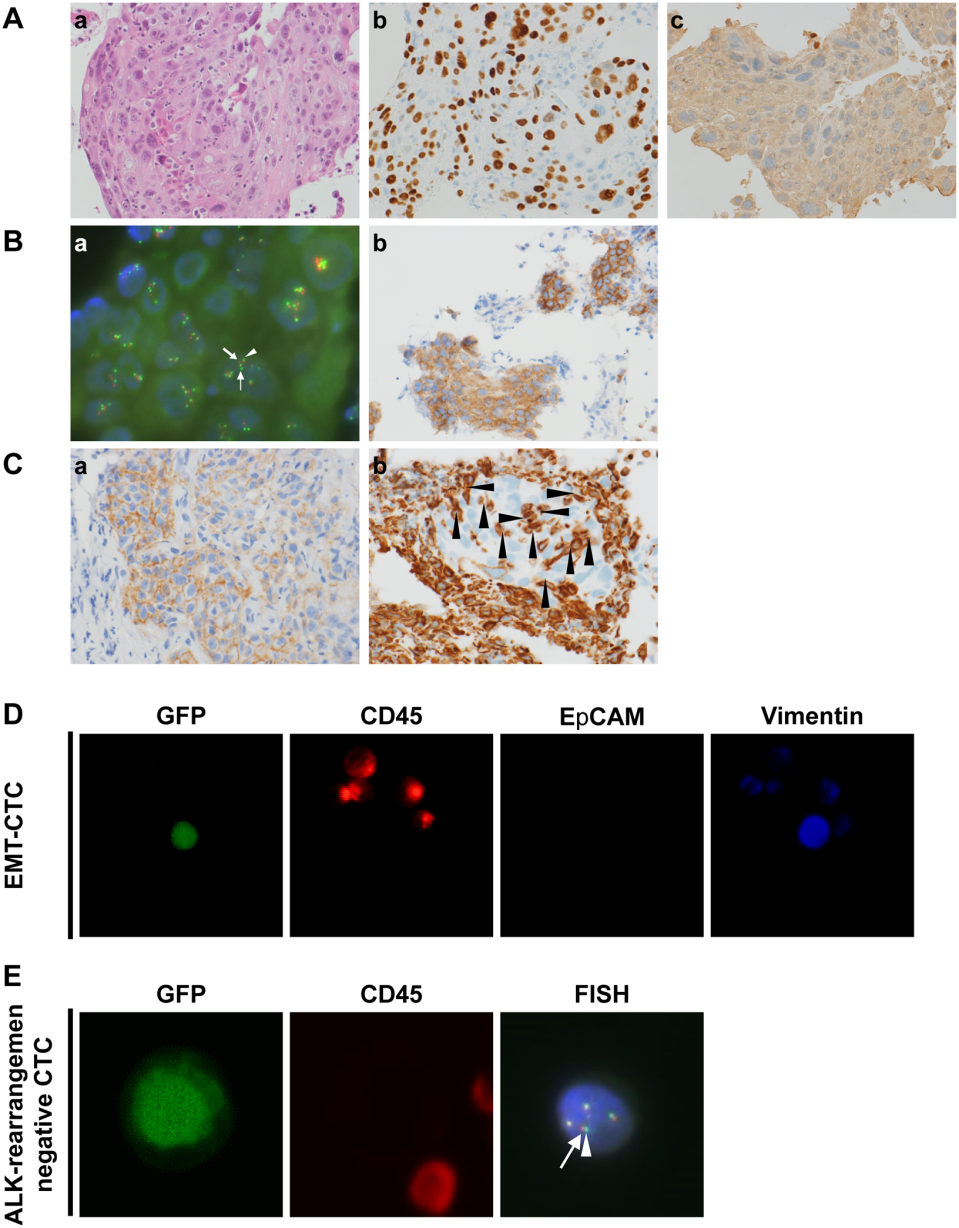
Immunohistological characteristics of case 1, a responder to ALK inhibitors **(A)** Immunohistochemical analysis of lung cancer tissue. a, Hematoxylin and eosin staining (100×); b, positive staining for p40 (100×); c, negative staining for TTF-1 (100×). **(B)** Diagnosis of ALK rearrangement. a, FISH analysis. Nuclei show a split positive pattern with separation between the 5' ALK green part (small arrow) and 3' ALK orange part (large arrow) of the FISH probe signal, indicating ALK rearrangement. Close apposition of the 3'/5' ALK parts (arrowhead) can be seen as a pseudo-colored (yellow) signal. b, Immunohistochemical analysis of ALK protein expression in tumor cells; ALK protein was overexpressed (IHC score 3) (100×). **(C)** Immunohistochemical analysis of off-target markers for ALK inhibitor resistance by rebiopsy of lung cancer tissue at the time of relapse after previous ALK inhibitor treatment. a, Positive staining for EGFR (150×); b, positive staining for vimentin in cancer cells surrounded by cancer stroma (arrowheads) (150×). **(D)** EMT-CTCs detected by liquid biopsy at the time of relapse after previous ALK inhibitor treatment. CTCs were positive for GFP using the telomerase-specific replication-selective adenovirus TelomeScan. False-positive cells were identified based on CD45 expression status. EMT CTCs were identified as GFP^+^/CD45^−^/EpCAM^−^/vimentin^+^. **(E)** ALK-rearranged CTCs identified by liquid biopsy at the time of relapse after previous ALK inhibitor treatment. CTCs (GFP^+^/CD45^−^) in blood samples with confirmed ALK rearrangement were subjected to FISH analysis. Close apposition of the 3' ALK orange part (arrow) and 5' ALK green part (arrow head) of the FISH probe signal indicates an intact wild-type copy of ALK in the cells.

**Figure 2:**
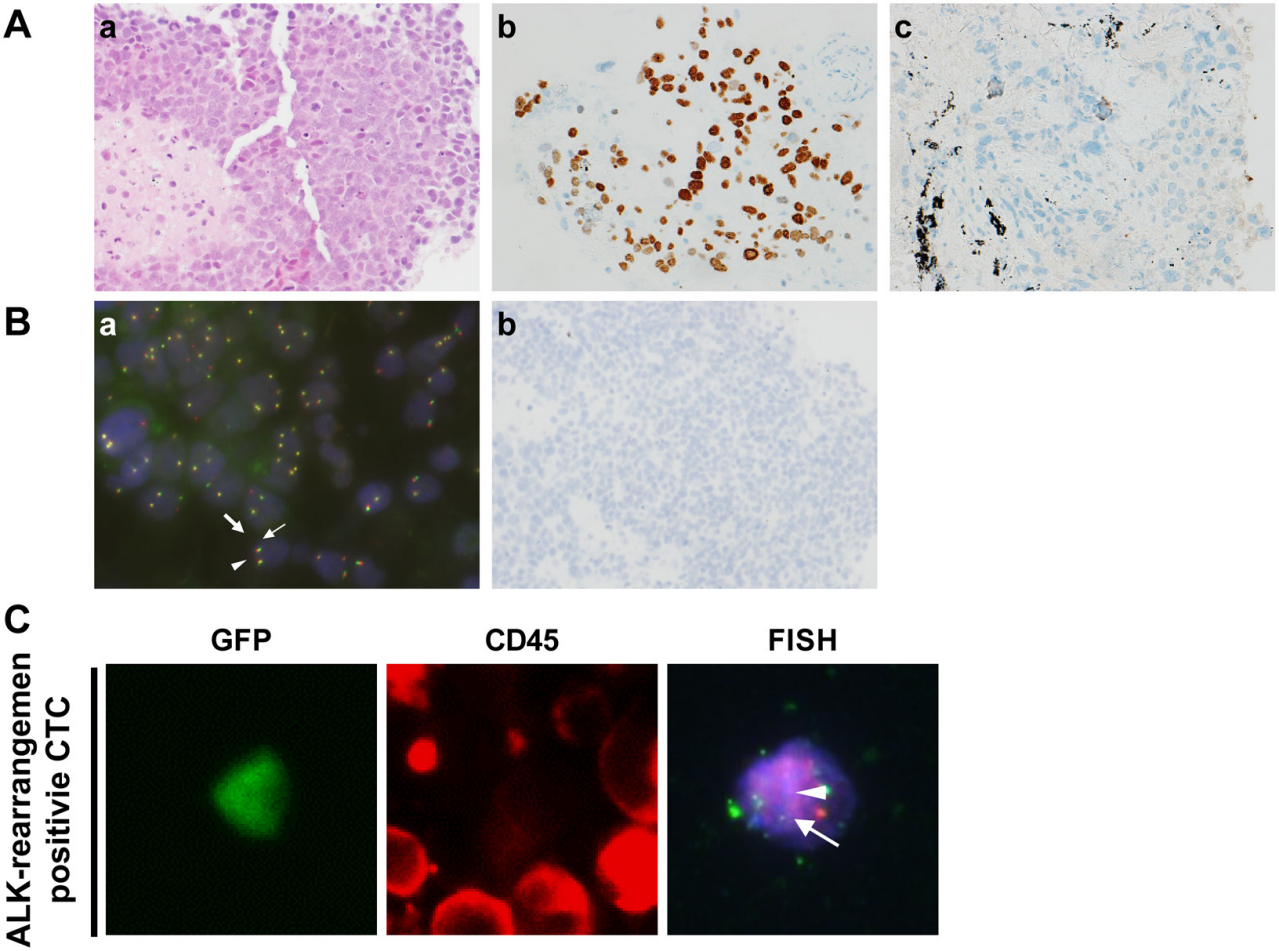
Immunohistological characteristics of case 2, a non-responder to ALK inhibitors **(A)** Immunohistochemical analysis of lung cancer tissue. a, Hematoxylin and eosin staining (100×); b, positive staining for p40 (100×); c, negative staining for TTF-1 (100×). **(B)** Diagnosis of ALK rearrangement. a, FISH analysis. Nuclei show a split positive pattern with separation between 5' ALK green part (small arrow) and 3' ALK orange part (large arrow) of the FISH probe signal, indicating ALK rearrangement. Close apposition of the 3'/5' ALK part (arrowhead) can be seen as a pseudo-colored (yellow) signal. b, Immunohistochemical analysis of ALK protein expression in tumor cells; ALK protein was not expressed. **(C)** ALK rearrangement-positive CTCs identified by liquid biopsy at the time of relapse following previous ALK inhibitor treatment. CTCs were positive for GFP using TelomeScan F35 and false-positive cells were identified based on their CD45 expression status (GFP^+^/CD45^−^). GFP-positive CTCs in blood samples with confirmed ALK rearrangement were subjected to FISH analysis. The nuclei showed a split positive pattern with separation between the 3' ALK orange part (arrow) and 5' ALK green part (arrowhead) of the FISH probe, consistent with ALK rearrangement.

Sq-LC showed typical histopathological features, including eosinophilic foci of intracellular keratinization and intercellular bridges around tumor cells (Figures 1A and 2A). All five cases were positive for both p63 and p40 expression. Cancer cells in the pleural effusion of case 1 were immunopositive for thyroid transcription factor (TTF)-1, while tumor tissue in case 5 was positive for TTF-1. Therefore, these two patients were provisionally diagnosed with ALK-rearranged adenosquamous cell (AdSq)-LC. All five cases were ALK-positive by FISH; however, two were negative by IHC (cases 2 and 4; Table 2). The rate of ALK immunopositivity by IHC was lower in Sq-LC than in Ad-LC with ALK rearrangement (60% and 84%, respectively; Tables 1 and 3).

**Table 2:** Clinicopathological features of patients with squamous and adenosquamous cell lung carcinoma with ALK rearrangement and comparisons of PFS from previous clinical studies

Present cases^*^	Age/ sex	Smoking history	Cancer type	Stage	IHC for Sq diagnosis				ALK-rearrangement detection			ALK inhibitor					
		PY	Sq or AdSq		p40	p63	TTF−1	Napsin A	IHC	% Positive FISH		First regimen	PFS (mo)	Second regimen	PFS (mo)	Third Regimen	PFS (mo)
**CASE 1**	64/F	0	AdSq^a^	IV	+	+	−	−	+	+	94	Crizotinib	7	Alectinib	4	Ceritinib	8
**CASE 2**	65/F	25	Sq	IV	+	+	−	−	−	+	78	Crizotinib	2	Alectinib	1	Ceritinib	0.5
CASE 3	36/M	23	Sq	IIIB	+	+	−	N/A	+	+	86	Crizotinib	12	Alectinib	5	N/A	N/A
CASE 4	62/M	21	Sq	IV	+	+	−	N/A	−	+	21	Crizotinib	1	N/A	N/A	N/A	N/A
CASE 5	47/M	20	AdSq	IV	+	+	+	N/A	+	+	66	Crizotinib	9	N/A	N/A	N/A	N/A
												Mean PFS	6.2	Mean PFS	3.3		
Clinical studies																	
PROFILE1007 [2]												Crizotinib	7.7				
PROFILE1014 [3]												Crizotinib	10.9				
AF−001JP [4]														Alectinib	>29		
NP28673 [5]														Alectinib^b^	8.9		
ASCEND−2 [6]																Ceritinib^b^	5.7
ASCEND−3 [7]																Ceritinib	13.8

AdSq, adenosquamous; ALK, anaplastic lymphoma kinase; F, female; FISH, fluorescence *in situ* hybridization; IHC, immunohistochemistry; M, male; mo, month; N/A, not available; PFS, progression-free survival; PY, pack year; Sq, squamous.

^a^Cytology from pleural effusion showed lung adenocarcinoma (p40^−^/p63^−^/TTF-1^+^).

^b^After crizotinib resistance.

^*^Cases 4 and 5 were added from Juntendo Urayasu Hospital.

### Detection of neoplastic nuclei by ALK FISH in ALK-rearranged Sq-LC

**Figure 3:**
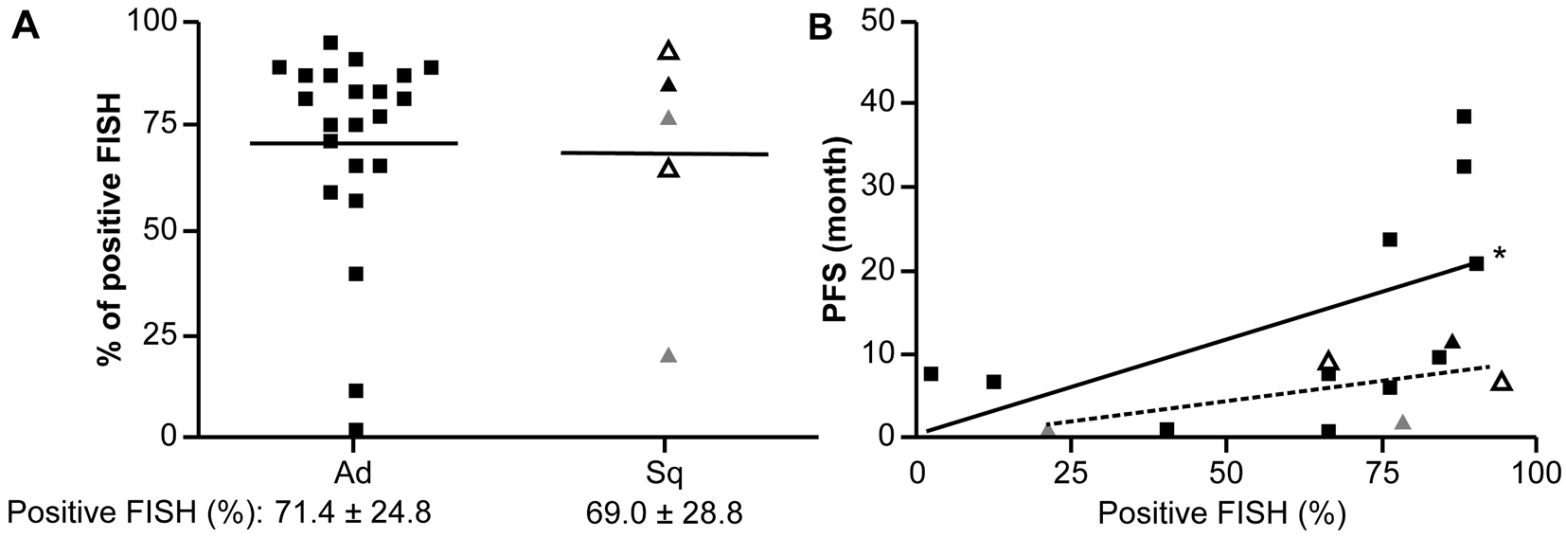
Rate of neoplastic nuclei detected by ALK FISH in ALK-rearranged Sq-LC and PFS following treatment with ALK inhibitors **(A)** Comparison of rates of neoplastic nuclei between ALK-rearranged Sq-LC and Ad-LC, as determined by ALK FISH. **(B)** Comparison of relationships between rates of neoplastic nuclei (detected by ALK FISH) and PFS following treatment with initial ALK inhibitors in ALK-rearranged Sq-LC and Ad-LC. Squares indicate ALK-rearranged Ad-LC; open triangles indicate AdSq-LC; triangles indicate ALK-rearranged Sq-LC; and gray triangles indicate patients with ALK-rearranged Sq-LC who were FISH-positive/IHC-negative. Each symbol represents an individual patient. ^*^P < 0.10, positive relationship between PFS and rate of neoplastic nuclei in the ALK-rearranged Ad group.

All patients with ALK-rearranged Sq-LC were subjected to ALK FISH analysis. There was no significant difference in the frequency of neoplastic nuclei between Sq-LC and Ad-LC with ALK rearrangement (69.0% ± 28.8% vs. 71.4% ± 24.8; Figure 3A). The frequency of neoplastic nuclei by ALK FISH tended to be positively correlated with progression-free survival (PFS) and associated with initial ALK inhibitor response in patients with ALK-rearranged Ad-LC (R^2^ = 0.300: *p* = 0.081); however, only a weak association was observed in patients with ALK-rearranged Sq-LC (*p* = 0.284), although the number of patients was too low for statistical analysis (Figure 3B).

### Clinical outcomes of patients with ALK-rearranged Sq-LC treated with ALK inhibitors

**Figure 4:**
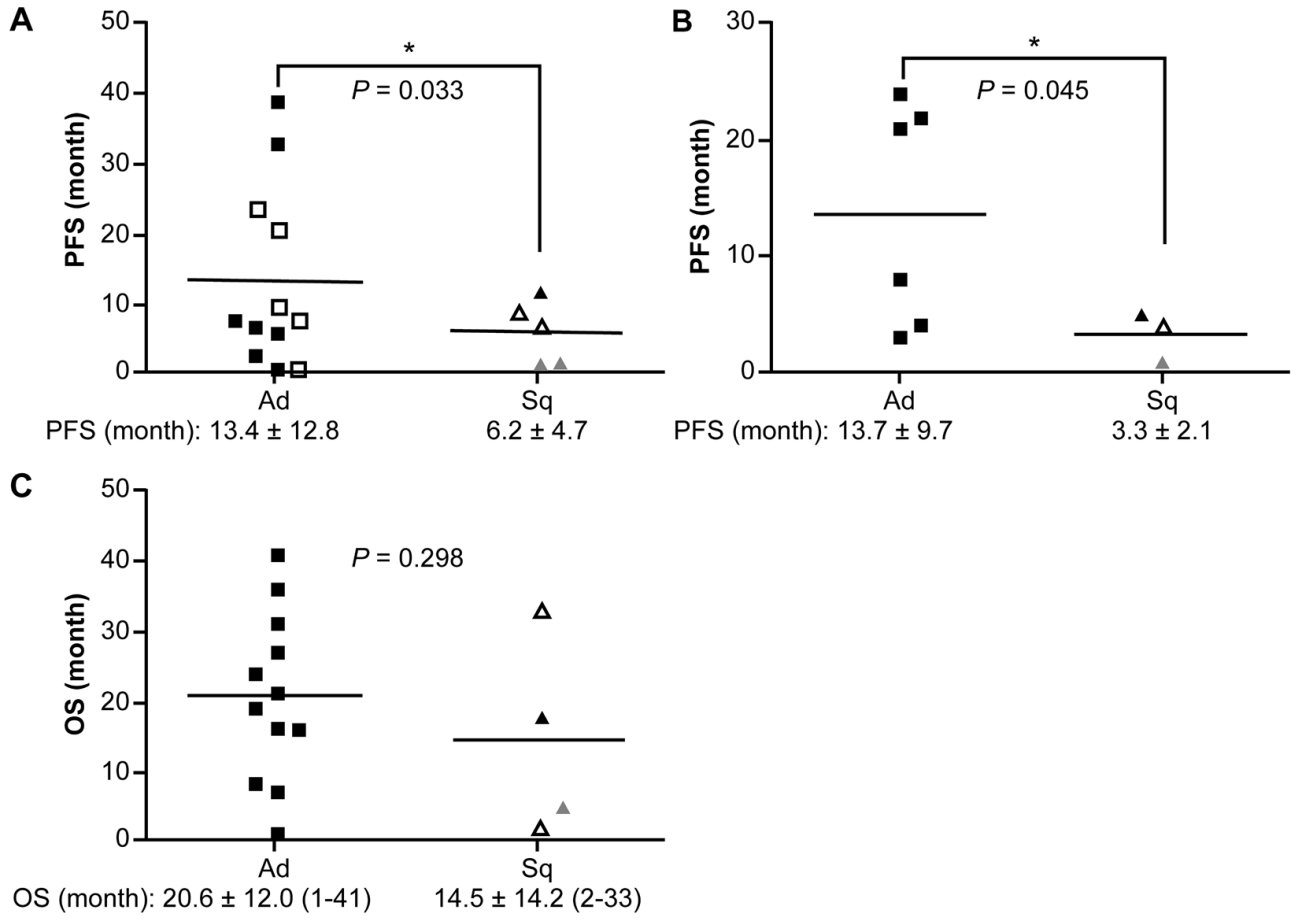
PFS and OS in patients treated with ALK inhibitors Comparisons of PFS between ALK-rearranged Sq-LC and Ad-LC patients following treatment with ALK inhibitors. **(A)** Initial treatment with ALK inhibitors. **(B)** Alectinib administration as second-line therapy. **(C)** Comparison of OS between ALK-rearranged Sq-LC and Ad-LC groups following treatment with ALK inhibitors. Vertical axis: duration of PFS or OS. Open triangles indicate AdSq-LC; open squares indicate patients who received alectinib as the initial ALK inhibitor; and gray triangles indicate patients with ALK-rearranged Sq-LC who were FISH-positive/IHC-negative. Each symbol represents an individual patient. ^*^P < 0.05 vs. Ad-LC group.

All patients with ALK-rearranged Sq-LC were treated with the ALK inhibitor crizotinib as second-line chemotherapy after a heterogeneous history of treatment with standard chemo- or chemo-/radiotherapy for Sq-LC. Accordingly, the median PFS of ALK-rearranged Sq-LC was shorter than that of ALK-rearranged Ad-LC (6.2 ± 4.7 vs. 13.4 ± 12.8 months, respectively; *p* = 0.033; Figure 4A). Notably, two cases of ALK-rearranged pure Sq-LC that were negative by IHC (cases 2 and 4) had an extremely short PFS; however, they did not differ in terms of PFS from two cases of ALK-rearranged AdSq-LC (cases 1 and 5) (Figure 4A and Table 2). Furthermore, patients with ALK-rearranged Sq-LC who received the ALK inhibitor alectinib as second-line chemotherapy were non-responsive and had extremely short PFS with PD as compared to patients with ALK-rearranged Ad-LC (3.3 ± 2.1 vs. 13.7 ± 9.7 months; *p* = 0.045; Figure 4B). The OS of patients with ALK-rearranged Sq-LC was almost 6 months shorter than that of patients with ALK-rearranged Ad-LC (14.5 ± 14.2 [2–35] vs. 20.6 ± 12.0 [1–44] months, respectively; *p* = 0.298), suggesting that outcomes are worse for the former as compared to the latter patient group even when ALK inhibitors are used (Figure 4C).

### Representative cases of responders and non-responders to ALK inhibitors

#### Case 1: an ALK inhibitor responder

A 64-year-old Japanese woman without a history of smoking was referred to Juntendo University Hospital. The patient was clinically diagnosed with NSCLC (cT4N3M1b, stage IV). A histopathological analysis of biopsied tumor tissue revealed Sq-LC without an adenocarcinoma component. IHC confirmed positivity for p40 and p63 and negativity for TTF-1 and napsin A; however, an analysis of cancer cells from plural effusion showed adenocarcinoma, with positive staining for TTF-1. FISH and IHC confirmed ALK rearrangement during the first cycle of first-line chemotherapy with a combination of nab-paclitaxel and carboplatin, which did not suppress the rapid growth of the tumor (i.e., PD). The chemotherapy regimen was interrupted and replaced with crizotinib. The patient showed a partial response but relapsed after 7 months. Stable disease was achieved following administration of alectinib. After 3 months, the tumor relapsed, and we rebiopsied the primary tumor nodule. The histological type was still Sq-LC, and ALK rearrangement was detected by IHC and FISH. The rebiopsied tissue was also evaluated for expression of EGFR and vimentin—a marker of epithelial-to-mesenchymal transition (EMT)—by IHC and for the presence of secondary mutations conferring resistance to ALK inhibitors by real-time (RT-PCR). The specimens were positive for EGFR and vimentin as well as for L1196M, a gatekeeper mutation conferring resistance to ALK inhibitors except for the second-generation inhibitor ceritinib. We therefore attempted ceritinib treatment; this increased PFS, with the patient showing a complete response. A liquid biopsy of circulating tumor cells (CTCs) was also performed, and EMT-CTCs positive for vimentin but negative for ALK rearrangement were detected per 7.5 ml blood by FISH (Figure 1).

#### Case 2: an ALK inhibitor non-responder

A 65-year-old Japanese woman who was a current smoker was referred to Juntendo University Hospital. The patient was clinically diagnosed with NSCLC (cT4N2M1b, stage IV). IHC analysis of biopsied tumor tissue revealed positive p40 and p63 and negative TTF-1 and napsin A immunoreactivity, confirming a diagnosis of Sq-LC. Carboplatin plus tegafur/gimeracil/oteracil was administered as first-line chemotherapy. However, this regimen was discontinued due to severe adverse effects, although there was a reduction in tumor size. We confirmed by FISH that the tumor harbored an ALK rearrangement, but the ALK-IHC score was zero during chemotherapy, and crizotinib was administered as second-line chemotherapy; after 8 weeks, the disease had progressed. Alectinib was administered but the tumor further progressed after 4 weeks (i.e., PD). We performed a non-invasive liquid-biopsy for cell-free (cf) DNA and CTCs instead of an invasive rebiopsy because the patient’s condition had rapidly deteriorated. ALK rearrangement was still present, but no secondary mutations conferring ALK inhibitor resistance were detected by RT-PCR, and three of 15 CTCs were still ALK rearrangement positive by FISH. We therefore co-administered the ALK-selective inhibitor ceritinib. However, the primary and metastatic lesions rapidly progressed after 2 weeks and the treatment course was changed to palliative in-home care (Figure 2).

## DISCUSSION

This is the first retrospective study comparing advanced Sq-LC and Ad-LC with ALK rearrangements based on clinical features and response to ALK inhibitors. Our findings—including from two case studies—provide important insights into therapeutic outcome that can guide clinicians in making decisions regarding the appropriate therapeutic course. Our retrospective analysis of ALK-rearranged Sq-LC revealed that ALK-rearranged Sq-LC may occur as a result of unexpected biological events and independent of ALK rearrangement due to the heterogeneity of the cancer.

The echinoderm microtubule-associated protein-like (EML) 4–ALK-positive rate depends on tissue size and is higher in surgical as compared to small biopsy specimens [8]. In our study, the prevalence of ALK rearrangement in patients with Sq-LC was 1.36%; although all of the cases were diagnosed using small biopsy specimens, this rate was consistent with previous reports [9, 10]. One study found that three of 207 (1.4%) cases of Sq-LC were ALK positive, as detected by IHC; this was confirmed by ALK FISH and RT-PCR [16]. Screening for ALK rearrangement in Sq-LC patients by FISH and/or IHC is not widespread due to its low prevalence. However, patients with ALK-rearranged Sq-LC showed poor clinical outcomes and account for 10.7% of all patients with NSCLC harboring ALK rearrangement; therefore, this form of LC cannot be dismissed.

p63 is an Sq-LC marker that is frequently co-expressed with TTF-1 and is often used to diagnose ALK-rearranged Ad-LC [17]. However, p40 is a more reliable marker than p63 for detecting Sq-LC with high specificity [18]. In the present study, all five ALK-rearranged Sq-LC cases were double-positive for p63 and p40. Furthermore, upregulation of the adenocarcinoma-specific marker SLX was detected in all cases with elevated levels of CYFRA, a Sq-LC-specific marker. These similarities in the clinical manifestations of ALK-rearranged Sq-LC and Ad-LC suggest that the tumor may exhibit heterogeneity, including an adenocarcinoma component (e.g., AdSq-LC) with ALK rearrangement, as detected in our cases and those that were previously described. Therefore, we could not definitively exclude the possibility of mixed squamous and adenocarcinomatous components even though the single small biopsy sample showed features of Sq-LC only. Some cases were actually identified as ALK-rearranged AdSq-LC but did not exhibit prolongation of PFS or improved OS in response to ALK inhibitor treatment, as observed in ALK-rearranged pure Ad-LC. Previous studies have reported that LC patients diagnosed with adenocarcinoma harboring EML4–ALK rearrangement tended to be younger and never or light smokers [20, 21]. In the present study, four of five (80%) patients were current or ex-smokers; thus, ALK-rearranged Sq-LC arising in the context of smoking history can aid in the identification of this rare type of LC.

Some patients with ALK-positive Sq-LC who were never smokers or light ex-smokers responded to crizotinib and showed a PFS longer than 5.8 months [22–26]. However, even these temporary responders to crizotinib showed shorter PFS than those in previous clinical trials of ALK-rearranged Ad-LC (PROFILE1007: 7.7 months and PROFILE1014: 10.9 months). A 76-year-old elderly man with a heavy smoking history who was diagnosed with ALK-rearranged Sq-LC showed a low percentage of neoplastic nuclei by ALK FISH (20%) and immunonegativity by IHC [21]. In the present study, the two patients with ALK-rearranged Sq-LC (cases 2 and 4) were older than 60 years with a history of heavy smoking, and case 4 had the lowest rate of rearrangement-positive cells (21%); moreover, they harbored ALK rearrangement in FISH-positive/IHC-negative tumors and did not respond to ALK inhibitors, with median PFS and OS of 1.8 and 5.3 months, respectively. The rate of discordance between ALK rearrangement detection by FISH and IHC ranges from 0.3% to 2% [27]. Five cases showing such discordance and lacking c-MET expression had lower and borderline rearrangement-positive cell rates of 15.0%–20.0%, and two did not respond to the dual ALK/c-MET inhibitor crizotinib [28]. ALK rearrangement in FISH-positive/IHC-negative patient-derived xenograft models also exhibited resistance to crizotinib [29]; the median percentage of neoplastic nuclei by ALK FISH was 35% [30].

The ALK rearrangement in FISH-positive/IHC-negative patient with ALK-rearranged Sq-LC (case 2) responded to standard chemotherapy for Sq-LC, which was replaced by ALK inhibitors due to adverse effects. This patient did not respond even to the more selective inhibitor ceritinib, despite the persistence of ALK rearrangement-positive cfDNA without secondary mutations conferring ALK inhibitor resistance and ALK rearrangement by FISH-positive CTCs.

The low rate of ALK rearrangement positivity by FISH—as evidenced by the low correlations between the rates of neoplastic nuclei by FISH and PFS following initial ALK inhibitors in ALK-rearranged Sq-LC, and by IHC negativity (cases 2 and 4) suggest that tumor tissues from patients with ALK-rearranged Sq-LC may contain heterogeneous tumor components including the original Sq-LC without ALK rearrangement and an independent oncogene-addiction phenotype, which are attributable to EML4–ALK mutations

Alectinib is safer and more effective than crizotinib for the treatment of ALK- rearranged LC [4]. One study described the case of a 78-year-old male ex-smoker with ALK- rearranged Sq-LC who did not respond to alectinib, although the tumor cells were found to be diffusely and strongly positive for ALK rearrangement by IHC and FISH [31]. Notably, none of the cases in our study that relapsed following initial treatment with the ALK inhibitor crizotinib responded to alectinib. Bypassing activation of the growth factor receptor loop is an off-target mechanism underlying the acquisition of ALK inhibitor resistance. In general, EGFR is expressed at higher levels in Sq-LC than in non-Sq-LC as determined by IHC analysis, and is co-expressed with ligands for EGFR such as TGF-α or IGF1R, leading to poor survival in NSCLC [32–34]. Case 1 exhibited high expression of EGFR in tumor tissue after showing no response to alectinib. Increased phosphorylation of EGFR is often detected in alectinib-resistant cancer cells, and TGF-α blockade occurs in NSCLC with restored sensitivity to alectinib [15]. Thus, EGFR and its ligands may have critical roles in ALK-rearranged Sq-LC as an off-target mechanism of alectinib resistance. On the other hand, a case of successful management of ALK-rearranged Sq-LC by initial treatment with alectinib has been reported [19], although additional cases must be studied in order to determine whether this is a general phenomenon.

Various secondary mutations have been identified following acquired resistance to current ALK inhibitors. L1196M was detected in a rebiopsied tumor tissue sample from case 1 who showed resistance to both crizotinib and alectinib and significantly prolonged PFS in response to ceritinib, along with immunopositivity for EGFR and EMT markers. L1196M is an on-target secondary mutation conferring resistance to ALK inhibitors and was present in only 7% of all of crizotinib-resistant specimens. L1196M-mutant cancer cells show the greatest response to ceritinib; the half-maximal inhibitory concentration of L1196M-overexpressing cancer cells is 339 nM for crizotinib, > 117.6 nM for alectinib, > 34.0 nM for loratinib, > 26.5 nM for brigatinib, and 9.3 nM for ceritinib [13]. P-glycoprotein (P-gp/ATP-binding cassette sub-family B member 1) exports ceritinib and contributes to ceritinib and crizotinib resistance. However, alectinib is unrelated to P-gp-mediated resistance mechanisms [35]. Thus, it is speculated that the resistance caused by secondary on-target ALK mutations rather than off-target activity underlie oncogenic addiction in this case.

EMT, as evidenced by reduced expression of the epithelial marker E-cadherin and upregulation of the mesenchymal marker vimentin [36], is thought to confer resistance to crizotinib in ALK-rearranged NSCLC [37]. In case 1, vimentin was expressed in both tumor tissues and CTCs, suggesting that EMT led to off-target resistance to ALK inhibitor. However, ceritinib was effective, suggesting that it potently inhibits ALK rearrangement mediated signal even in the presence of EMT, and thus EMT may not be directly required for ALK inhibitor resistance. Sensitivity to ceritinib was restored in cancer cells that had undergone EMT and acquired resistance to ceritinib after a drug holiday, suggesting that EMT-mediated resistance is partly reversible and that ALK mutation drives oncogenic addiction [38]. ALK inhibitors also directly inhibit EMT via upregulation of epithelial splicing regulatory protein 1 (ERSP1) [39]. Therefore, although there was evidence of EMT in rebiopsied tumor samples in the absence of an ALK inhibitor response, administration of additional ALK inhibitors can be effective.

Although sample sizes in the present study were small and nearly all information regarding clinical outcomes was derived from retrospective studies, oncologists should consider the possibility of ALK-rearranged Sq-LC by recognizing its clinicopathological features. ALK inhibitors still represent a reasonable option for treating ALK-rearranged Sq-LC instead of the standard chemotherapy used for Sq-LC, although patient outcome is worse than in ALK-rearranged Ad-LC patients. Furthermore, standard chemotherapy for Sq-LC should be considered as a therapeutic option for patients with ALK-rearranged Sq-LC who harbor ALK rearrangement in FISH-positive/IHC-negative tumors and do not respond to ALK inhibitors. Recognizing the complexity of on- and off-target resistance mechanisms to ALK inhibitors in ALK-rearranged Sq-LC through analysis of aggressive rebiopsy samples can provide important information regarding the efficacy of second-line ALK inhibitors and improve clinical outcome. Additional large-scale clinical studies that do not depend on individual case reports are needed to clarify the response to ALK inhibitors and on- and off-target resistance mechanisms in the rare cases of ALK-rearranged Sq-LC.

## MATERIALS AND METHODS

### Ethics statement

This investigation was conducted in accordance with the ethical standards outlined by the Declaration of Helsinki and according to national and international guidelines. The protocol was approved by the institutional review boards of Juntendo University and Kenshokai Fukushima Healthcare Center, affiliated with Oncolys BioPharma Inc. Written, informed consent was obtained from patients for publication of case reports.

### Study design

We retrospectively analyzed the clinical features of five patients with ALK-rearranged Sq-LCs, including two who were confirmed as having an adenocarcinoma component (cases 1 and 5). To analyze the prevalence of ALK-rearranged Sq-LC, three of 28 patients with ALK-rearranged Ad-LC were enrolled at Juntendo University Hospital from November 2011 to March 2017. We routinely evaluated all NSCLC patients including those with Sq-LC for ALK rearrangement by both ALK FISH and IHC. Two additional patients from Juntendo Urayasu Hospital were enrolled for statistical analysis. The clinical features of the five patients with ALK-rearranged Sq-LC were analyzed and compared with those of patients with ALK-rearranged Ad-LC. The maximal response to ALK inhibitors was monitored by radiologic assessment at least every 4 weeks after initial treatment based on Response Evaluation Criteria in Solid Tumors (RECIST) v.1.1 [46].

### Histopathological and immunohistochemical analyses

Tissue samples were fixed in 10% buffered formalin, embedded in paraffin after routine processing, and cut into sections that were stained with hematoxylin and eosin. IHC was performed with the EnVision+System (Dako Cytomation, Carpinteria, CA, USA) using mouse monoclonal antibodies against TTF-1 (8G7G3/1, 1:200 dilution; Dako Cytomation), p40 (BC28, 1:100 dilution; Biocare Medical, Pacheco, CA, USA), p63 (BC28, 1:100 dilution; Biocare Medical), and vimentin (V9, 1:800 dilution; Leica Biosystems, Nussloch, Germany).

### Analysis of secondary mutations conferring ALK inhibitor resistance

For DNA extraction, samples were placed in a sterile tube with lysis buffer (Promega, Madison, WI, USA) and were mechanically disrupted using TissueLyser TL (Qiagen, Venlo, The Netherlands) for 1 min at 50 Hz. Proteinase K (Promega) and RNase A solution (Sigma-Aldrich, St. Louis, MO, USA) were added to the samples followed by vortexing (Scientific Industries, Bohemia, NY, USA) and overnight incubation at 56°C. DNA isolation was performed using a Maxwell16 Blood DNA Purification kit on a Maxwell16 Instrument (Promega) according to manufacturer’s instructions. DNA was eluted in 40 μl nuclease-free water, and concentration and purity were measured using an Agilent 2200 TapeStation with Genomic DNA ScreenTape (Agilent Technologies, Santa Clara, CA, USA). Briefly, digital PCR was performed using a QX200 Digital Droplet PCR platform (Bio-Rad Laboratories, Hercules, CA, USA). Reactions were set up with Probe Supermix (Bio-Rad Laboratories) and LBx probes (RIKEN GENESIS Co., Tokyo, Japan); ALK Multi1 (A089), ALK Multi2 (A090), and ALK Multi3 (A091) were used for mutation screening, and ALK T1151ins (A085), ALK C1156Y (A067), ALK L1196M (A071), and ALK G1269A (A074) were used for single typing verification. Thermal cycling was performed on a Veriti instrument (Applied Biosystems, San Mateo, CA, USA) according to the following protocol: 10 min at 95°C; 40 cycles of 30 s at 94°C and 1 min at 58°C; and 10 min at 98°C with a 50% ramp rate. Results were analyzed using QuantaSoft v.1.6.6.0320 software (Bio-Rad Laboratories).

### Analysis of CTCs

Blood samples (7.5 ml) were collected from patients with ALK-rearranged Sq-LC and sent to the Clinical Laboratory Center, Oncolys BioPharma for analysis of CTCs. Red blood cells were lysed and white blood cells were isolated. The cell pellet was incubated with 1 × 10^9^ viral particle-modified adenovirus (OBP-1101; rAdF35-142T-GFP) for 24 h, and infected telomere-elongated CTCs were detected based on green fluorescent protein (GFP) signal intensity. Detected GFP-positive cells were immunolabeled with primary antibodies following fixation and permeabilization with 4% paraformaldehyde (cat. no. 09154-85; Nacalai Tesque, Kyoto, Japan) and 0.15% Triton X-100 (cat. no. 93343-100ML; Sigma-Aldrich), respectively. Primary antibodies against cluster of differentiation (CD) 45 (conjugated with Brilliant Violet 421) (304032, 3:100; BioLegend, San Diego, CA, USA), epithelial cell adhesion molecule (EpCAM) (ab7504, 1:100; Abcam, Cambridge, UK), and vimentin (ab45939, 1:200; Abcam) were used in this study. GFP-positive CTCs (GFP^+^/CD45^−^) were distinguishable from false-positive cells (GFP^+^/CD45^+^) by CD45 immunoreactivity. CTCs exhibiting mesenchymal features (i.e., EpCAM^−^/vimentin^+^) were defined as EMT CTCs. The experimental protocol has been previously described [47, 48].

### IHC and FISH analyses of ALK expression

IHC analysis of ALK in formalin-fixed, paraffin-embedded tumor tissue sections was performed according to a clinically optimized and standardized assay using a highly sensitive intercalated antibody-enhanced polymer method based on 5A4-Histofine staining (ALK Detection kit; Nichirei Bioscience, Tokyo, Japan) [4]. Samples were prospectively analyzed with the FDA-approved Vysis ALK Break-Apart FISH Probe kit (Abbott Molecular, Abbott Park, IL, USA) using an ALK break-apart (i.e., split-signal) probe [20]. Samples were deemed FISH-positive if > 15% of scored tumor cells had split ALK 5' and 3' probe signals or had isolated 3' signals. CTCs detected by OBP-1101 were fixed and collected on glass slides to visualize ALK rearrangement in the nuclei of GFP-positive cells. FISH was also performed using the Vysis ALK Break-Apart FISH Probe kit along with the Vysis Paraffin & Post-Hybridization Wash Buffer kit (Abbott Molecular) as previously described [49].

### Statistical analysis

Results are expressed as mean ± standard deviation. PFS and OS were compared between ALK-rearranged Sq-LC and Ad-LC groups with the unpaired Student’s t test, with each subject considered as an individual point. P values < 0.05 were considered significant. Data were analyzed using Prism 6 software (GraphPad Inc., San Diego, CA, USA).
